# Effect of Incorporating Oat Flour and Sourdough on the Sensory and Technological Characteristics of Bread

**DOI:** 10.1002/fsn3.4693

**Published:** 2025-01-06

**Authors:** Masoome Sayadi, Akram Arianfar, Ali Mohamadi Sani, Zahra Sheikholeslami

**Affiliations:** ^1^ Department of Food Science and Technology, Quchan Branch Islamic Azad University Quchan Iran; ^2^ Department of Agricultural Engineering Research, Khorasan Razavi Agricultural and Natural Resources, Research and Education Center AREEO Mashhad Islamic Republic of Iran

**Keywords:** bread, *Lactobacillus plantarum*, oat flour, sourdough

## Abstract

This study investigated the effects of different formulations on the technological and sensory properties of bread. The bread formulation included 9 variations of sourdough treatments and 4 variations of wheat flour and oat flour percentages. Results demonstrated that the highest increase in dough volume occurred in samples containing sourdough made from wheat, oat, *Lactobacillus plantarum* , and *Saccharomyces cerevisiae* at 64.5%–68%, and breads lacking yeast exhibited the lowest amount of porosity 2.3–7.2. Texture Profile Analysis (TPA) revealed that sourdoughs without *Sc. cerevisiae* exhibited significantly higher levels of hardness, chewiness, and cohesiveness. The stickiness of breads was most pronounced in those produced with wheat sourdough, *Sc. cerevisiae*, and *Lb. plantarum*. Conversely, samples containing wheat, oat, *Lb*. *plantarum*, and *Sc. cerevisiae* with varying wheat flour ratios (100%, 95%, and 85%) showed no stickiness. SEM analysis showed incorporating into bread with oat flour led to larger porosity. HunterLab measurements indicated that breads with sourdough of wheat, oat, *Lb. plantrum*, and *Sc. cerevisiae* had the highest values of parameters a*, b*, and Δ*E*, 11.2–11.7, 34.5–35.35, 0.52–0.58, respectively, while yeast‐free breads exhibited the highest L* value 72.3–72.9. Sensory evaluations indicated that breads produced with sourdough of wheat, oat, *Lb. plantarum*, and *Sc. cerevisiae* received the highest overall scores for taste, aroma, texture, appearance, and total acceptance from evaluators. Conversely, samples lacking yeast received the lowest scores. Therefore, the optimal bread formulation involved using sourdough composed of wheat, oat, *Lb. plantarum*, and *Sc. cerevisiae* with up to a 10% replacement of wheat flour with oat flour.

## Graphical abstract

Breads containing wheat, oat, *Lactobacillus plantarum* , and *Saccharomyces cerevisiae* showed the highest dough volume and best sensory properties. Breads without *Sc*. *cerevisiae* were harder, less springy, and darker in color. Sensory evaluations confirmed that the optimal formulation involved a 15% replacement of wheat flour with oat flour, yielding the highest taste, texture, and overall acceptance scores.

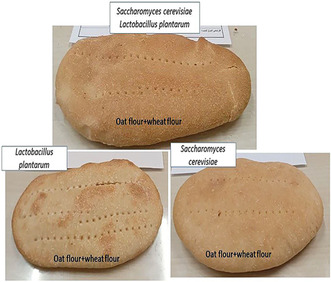

## 1 Introduction

Bread, as a primary food source, has traditionally been made using wheat flour. However, the global need for food is on the rise, particularly in developing countries where bread consumption is increasing. Unfortunately, these countries often face unfavorable climate conditions for wheat cultivation. This heavy reliance on wheat makes them more susceptible to disruptions in the wheat supply chain, whether due to unforeseen circumstances or human activities. Moreover, this dependence on wheat also has adverse effects on the environment and public health (Wang and Jian 2022). Wheat protein exhibits low levels of the essential amino acids lysine, valine, and tryptophan (Urošević et al. 2023). Compared to wheat protein, oat protein exhibits higher levels of lysine, threonine, tyrosine, and tryptophan. Additionally, oat protein contains soluble fractions of fiber that are rich in β‐glucans, with a concentration of up to 10 g per 100 g of dry matter (Krochmal‐Marczak, Tobiasz‐Salach, and Kaszuba 2020). Semi‐volume wheat bread, as the predominant bread produced in Iran, has a porous, spongy and uniform texture, and its thickness is between 2.5 and 5 cm (Arianfar et al. 2023). A fundamental issue in reducing the quality of bread lies in its spoilage, brought about by the physicochemical alterations in both the crust and crumb of the bread, leading to a decline in consumer acceptance (Chen 2020). Utilizing sourdough fermentation with targeted starter cultures presents an efficient method to diminish staleness and prolong the shelf life of bread (Hernández‐Figueroa et al. 2023). The utilization of sourdough in bread production has a rich historical background. Sourdough, a complex biological mixture, emerges from the fermentation of cereal flour or its constituents with water. It involves a symbiotic relationship between lactic acid bacteria and yeasts, which play pivotal roles in fermenting and acidifying the dough (Sadeghi 2008). Numerous investigations have been conducted regarding the microorganisms involved in the process of natural sourdough formation (Ewuoso, Animashaun, and Adejumo 2020). The most commonly encountered LAB in sourdough are *Lactobacillus sanfranciscensis* , *Lactobacillus brevis* , and *Lb. plantarum*, while *Sc. cerevisia* is the prevailing yeast strain (Minervini et al. 2012). Enhanced understanding of the technological function of lactic acid bacteria (LAB) in dough‐based products and their impact on *Sc. cerevisia* would prove highly beneficial in practice. By fostering favorable effects and mitigating unfavorable ones, the development of novel starters could be facilitated (Li et al. 2015). Sourdough alters the level of starch hydrolysis and diminishes the crystallization of starch, thereby mitigating bread staleness, through the regulation of flour's enzymatic activity (Nouska et al. 2023). Some of the metabolites produced by lactic acid bacteria in sourdough, including extracellular polysaccharides and their proteolytic and amylolytic enzymes, exhibit efficacy in retarding the occurrence of bread staleness (Dong 2022). Cera et al. (2024) explored the impact of sourdough, created using various lactic acid bacteria and yeast starters, on the texture and flavor improvement of oat bread (Cera et al. 2024). In another study, Huynh et al. (2024) assessed enhancing the nutritional value of bread by incorporating oat flour (5%, 15% and 25%) into wheat flour. Sensory evaluations indicated positive consumer responses, suggesting acceptance of oat‐enriched breads (Huynh et al. 2024).

Hence, the objective of the study was to examine the impact of *Sc. cerevisiae* and *Lb. plantarum* in the formulation of semi‐volume bread incorporating wheat flour and oat flour, and their influence on the rheological characteristics of the dough, sensory attributes, and shelf life of the final product.

## 2 Material and Methods

### 2.1 Strain and Materials

The fermentations involved LAB, namely, *Lb. plantarum* ATCC 8014 (*LP*) acquired from **Persian Type Culture Collection** and *Sc. cerevisiae* obtained from *Razavi* yeast. Co, Iran. For both microorganisms MRS agar and Sabaroud Dextrose Agar (Merck, Germany) was used, respectively. Wheat flour at the rate of extraction 85% was obtained from Kalaleh Flour Factory, Iran and uncoated oat flour was obtained from Baharan Company, Iran.

### 2.2 Dough Formulation

A total of nine formulations of sourdough were utilized (Table 1), incorporating 1 g of microorganisms (with different ratios of *Lb. plantarum* to *Sc. cerevisiae*, such as 0:1, 1:0, and 0.5:0.5), 100 g of flour (with varying ratios of wheat to oat flour, including 1:0, 0:1, and 0.5:0.5) and 60 g of water, prepared in one step for 5 min at room temperature. Additionally, Oat flour in ratios of 0%, 5%, 10%, and 15% of wheat flour was mixed with wheat flour and 60% warm water at 32°C–35°C, 0.6% sourdough, 0.4% salt, 0.4% sugar, and 1.15% oil was used. The ratios of all the ingredients used in the formulation are reported in proportion to the flour. In formulations that included oat flour, 0.2% guar gum was also added (Tables 2 and 3).

**TABLE 1:** Formulations of sourdough for bread making.

Formulation	Sample code	Wheat flour	Oat flour	*Lb. plantarum*	*Sc. cerevisia*
S1	W‐*Lb*	✓		✓	
S2	O‐*Lb*		✓	✓	
S3	W‐O‐*Lb*	✓	✓	✓	
S4	W‐*Sc*	✓			✓
S5	O‐*Sc*		✓		✓
S6	W‐O‐*Sc*	✓	✓		✓
S7	W‐*Sc*‐*Lb*	✓		✓	✓
S8	O‐*Lb*‐*Sc*		✓	✓	✓
S9	W‐O‐*Sc*‐*Lb*	✓	✓	✓	✓

**TABLE 2:** Semi‐bulky bread formulation.

Ingredient	% based on flour weight
Flour (wheat+oat)	100
Sourdough	0.6
Oil	1.15
Sugar	0.4
Salt	0.4
Water	60

**TABLE 3:** Different treatments of semi‐bulky bread.

Treatment	Sample code	Sourdough	Flour %
T1	W100/W—*Lb*	W—*Lb*	W100
T2	W100/O—*Lb*	O—*Lb*	W100
T3	W100/W‐O—*Lb*	W‐O—*Lb*	W100
T4	W100/W—*Sc*	W—*Sc*	W100
T5	W100/O—*Sc*	O—*Sc*	W100
T6	W100/W‐O—*Sc*	W‐O—*Sc*	W100
T7	W100/W—*Lb*—*Sc*	W—*Lb*—*Sc*	W100
T8	W100/O—*Lb*—*Sc*	O—*Lb*—*Sc*	W100
T9	W100/W‐O—*Lb*—*Sc*	W‐O—*Lb*—*Sc*	W100
T10	W95/O5/W—*Lb*	W—*Lb*	W95 + O5
T11	W95/O5/O—*Lb*	O—*Lb*	W95 + O5
T12	W95/O5/W‐O—*Lb*	W‐O—*Lb*	W95 + O5
T13	W95/O5/W—*Sc*	W—*Sc*	W95 + O5
T14	W95/O5/O—*Sc*	O—*Sc*	W95 + O5
T15	W95/O5/W‐O—*Sc*	W‐O—*Sc*	W95 + O5
T16	W95/O5/W—*Lb*—*Sc*	W—*Lb*—*Sc*	W95 + O5
T17	W95/O5/O—*Lb*—*Sc*	O—*Lb*—*Sc*	W95 + O5
T18	W95/O5/W‐O—*Lb*—*Sc*	W‐O—*Lb*—*Sc*	W95 + O5
T19	W90/O10/W—*Lb*	W—*Lb*	W90 + O10
T20	W90/O10/O—*Lb*	O—*Lb*	W90 + O10
T21	W90/O10/W‐O—*Lb*	W‐O—*Lb*	W90 + O10
T22	W90/O10/W—*Sc*	W—*Sc*	W90 + O10
T23	W90/O10/O—*Sc*	O—*Sc*	W90 + O10
T24	W90/O10/W‐O—*Sc*	W‐O—*Sc*	W90 + O10
T25	W90/O10/W—*Lb*—*Sc*	W—*Lb*—*Sc*	W90 + O10
T26	W90/O10/O—*Lb*—*Sc*	O—*Lb*—*Sc*	W90 + O10
T27	W90/O10/W‐O—*Lb*—*Sc*	W‐O—*Lb*—*Sc*	W90 + O10
T28	W85/O15/W—*Lb*	W—*Lb*	W85 + O15
T29	W85/O15/O—*Lb*	O—*Lb*	W85 + O15
T30	W85/O15/W‐O—*Lb*	W‐O—*Lb*	W85 + O15
T31	W85/O15/W—*Sc*	W—*Sc*	W85 + O15
T32	W85/O15/O—*Sc*	O—*Sc*	W85 + O15
T33	W85/O15/W‐O—*Sc*	W‐O—*Sc*	W85 + O15
T34	W85/O15/W—*Lb*—*Sc*	W—*Lb*—*Sc*	W85 + O15
T35	W85/O15/O—*Lb*—*Sc*	O—*Lb*—*Sc*	W85 + O15
T36	W85/O15/W‐O—*Lb*—*Sc*	W‐O—*Lb*—*Sc*	W85 + O15

Abbreviations: *Lb*, *Lactobacillus plantarum* ; O, oat flour; *Sc*, *Saccharomyces cerevisiae* ; W, wheat flour; W85 + O15, 85% wheat flour and 15% oat flour; W90 + O10, 90% wheat flour and 10% oat flour; W95 + O5, 95% wheat flour and 5% oat flour; W100, 100% wheat flour.

### 2.3 Bread Making

All ingredients mixed extensively using an industrial spiral bread mixer (Berjaya, Malaysia) for 10 min. Then, the dough was portioned into small pieces (200 g) and allowed to rest in a proofing cabinet (MIWE backcombi, Germany) for 20 min at 37°C and 75% relative humidity. Next, the doughs were spread in a length of 30 cm in a tray. The second fermentation lasted 40 min for samples containing *Sc. cerevisiae* and 4 h for those containing *Lb. plantarum* in a controlled environment with 75% relative humidity and a temperature of 37°C. Finally, they were baked in the oven (Forni, Verona, Italy) at 180°C for 15 min. The loaves were cooled at room temperature and then packed into polypropylene zip bags.

### 2.4 Physicochemical Properties of the Bread

The bread samples were assessed for their moisture, protein, and fiber content using the methodology outlined by the American Cereal Chemistry Association, 44–15, 46–12, 32–10. a_w_ was determined automatically by Novasina C‐500 AW‐LAB (Axair Ltd., Switzerland) (American Association of Cereal Chemists. Approved Methods Committee 2000; Miyazaki, Maeda, and Morita 2004). Enhancement in dough volume after secondary fermentation was calculated according to Equations (1) and (2). pH content was assessed using the methodology outlined by the American Cereal Chemistry Association 2–52 and specific volume was measured by AACC method number 55–50 (American Association of Cereal Chemists. Approved Methods Committee 2000). To perform Loaf specific volume test, AACC method number 55–50 was used (American Association of Cereal Chemists. Approved Methods Committee 2000).

(1) $$V={\text{34𝜋r}}^{2}h$$

where: r is the radius of dough piece, h is the height of dough piece.

(2) $$\Delta V=V_{\text{after}}-V_{\text{before}}$$

where: *V*_after_ is volume of dough after fermentation, *V*_before_ is volume of dough before fermentation.

### 2.5 Porosity

To assess the porosity of the bread crumb, an image processing technique was employed. Initially, a high‐resolution image (1200 pixels) of the bread crumb was generated using a scanner. Subsequently, these images were processed using software to convert them into gray‐level images by activating the 8‐bit mode.

### 2.6 Color Analysis

The evaluation of the color of bread crust was conducted by employing a Hunterlab ColorQuest colorimeter (ColorFlex EZ colorimeter, USA). Duplicate measurements were performed on three slices of each bread. The L* values indicated the brightness level on a scale ranging from 0 (dark) to 100 (white). Additionally, the +a* value represented the redness, while the −a* value indicated the greenness. Similarly, the +b* value denoted the yellowness, whereas the −b* value represented the blueness of the bread crust. Δ*E*, the color difference between the control sample and the test sample, was calculated according to the below equation:

(3) $$\Delta E=\surd {\left(\Delta L\right)}^{2}+{\left(\Delta a\right)}^{2}+{\left(\Delta b\right)}^{2}$$

### 2.7 TPA Analysis

The textural properties of the bread sample were assessed using a Texture Analyzer TVT‐6700 (Perten Instruments, Hägersten, Sweden). This texture analyzer was outfitted with a 10‐kg load cell to accurately measure the physical attributes of the bread such as hardness, cohesiveness, springiness, and chewiness. To conduct the analysis, the bread samples were sliced into 40‐mm and a thickness of 25 mm. A cylindrical probe (diameter 76.2 mm and length 10 mm) was applied at a controlled speed of 1 mm/s and a compression rate of 50% of the total height.

### 2.8 Microstructure of Bread

To analyze the microstructure of the bread, small pieces of bread crumb, approximately 5.2 mm in size, were cut from the central portion of the bread and were freeze‐dried (CHRist LD 1–4, Germany). The dried samples were then subjected to a gold vapor deposition process using a gold deposition machine (EMITECH K450X, UK) to make them conductive. Subsequently, images were captured in variable‐pressure mode at an accelerating voltage of 15 kV.

### 2.9 Sensory Characteristic

A 5‐point hedonic scale was employed for sensory assessments, wherein attributes such as appearance, odor, taste, texture, and overall acceptability were evaluated. The sensory attributes of the bread samples were assessed by a panel consisting of 20 judges who had received partial training in sensory evaluation techniques.

### 2.10 Statistical Analysis

The data were presented as the mean ± standard deviation. All experiments were performed in triplicate. Statistical analysis was conducted using the Statistical Package for Social Science (SPSS) software (version 16). A two‐way analysis of variance (ANOVA) with T‐test was applied to identify significant differences at a significance level of 5%.

## 3 Result and Discussion

### 3.1 Physicochemical analysis of Bread

**FIGURE 1:**
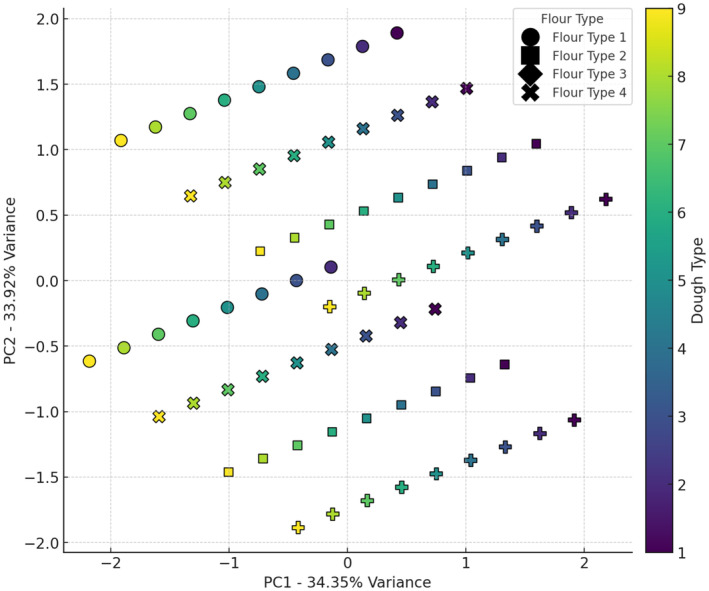
PCA analysis of the effect of different flour and sourdough combinations on bread moisture. Flour type: 1 (wheat), 2 (5% oat), 3 (10% oat), 4 (15% oat); Sourdough type: 1 (wheat + *L. plantarum* ), 2 (oat + *L. plantarum* ), 3 (wheat + oat + *L. plantarum* ), 4 (wheat + *Sc. cerevisiae*), 5 (oat + *Sc. cerevisiae*), 6 (oat + wheat + *Sc. cerevisiae*), 7 ( *L. plantarum* + *Sc. cerevisiae* + wheat), 8 (oat + *L. plantarum* + *Sc. cerevisiae*), 9 (wheat + oat + *L. plantarum* + *Sc. cerevisiae*).

**FIGURE 2:**
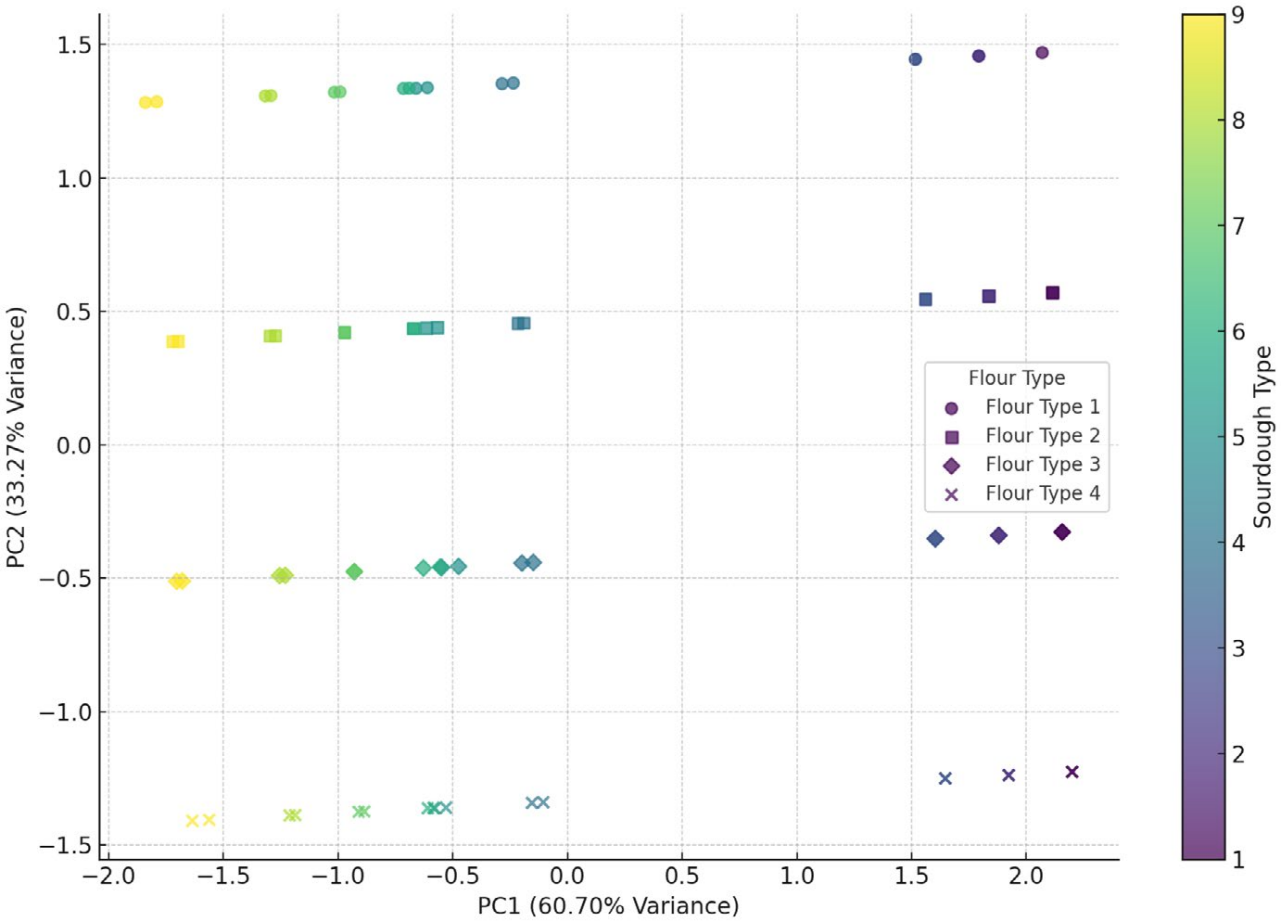
PCA analysis of the effect of different flour and sourdough combinations on enhancement of dough volume. Flour type: 1 (wheat), 2 (5% oat), 3 (10% oat), 4 (15% oat); Sourdough type: 1 (wheat + *L. plantarum* ), 2 (oat + *L. plantarum* ), 3 (wheat + oat + *L. plantarum* ), 4 (wheat + *Sc. cerevisiae*), 5 (oat + *Sc. cerevisiae*), 6 (oat + wheat + *Sc. cerevisiae*), 7 ( *L. plantarum* + *Sc. cerevisiae* + wheat), 8 (oat + *L. plantarum* + *Sc. cerevisiae*), 9 (wheat + oat + *L. plantarum* + *Sc. cerevisiae*).

**FIGURE 3:**
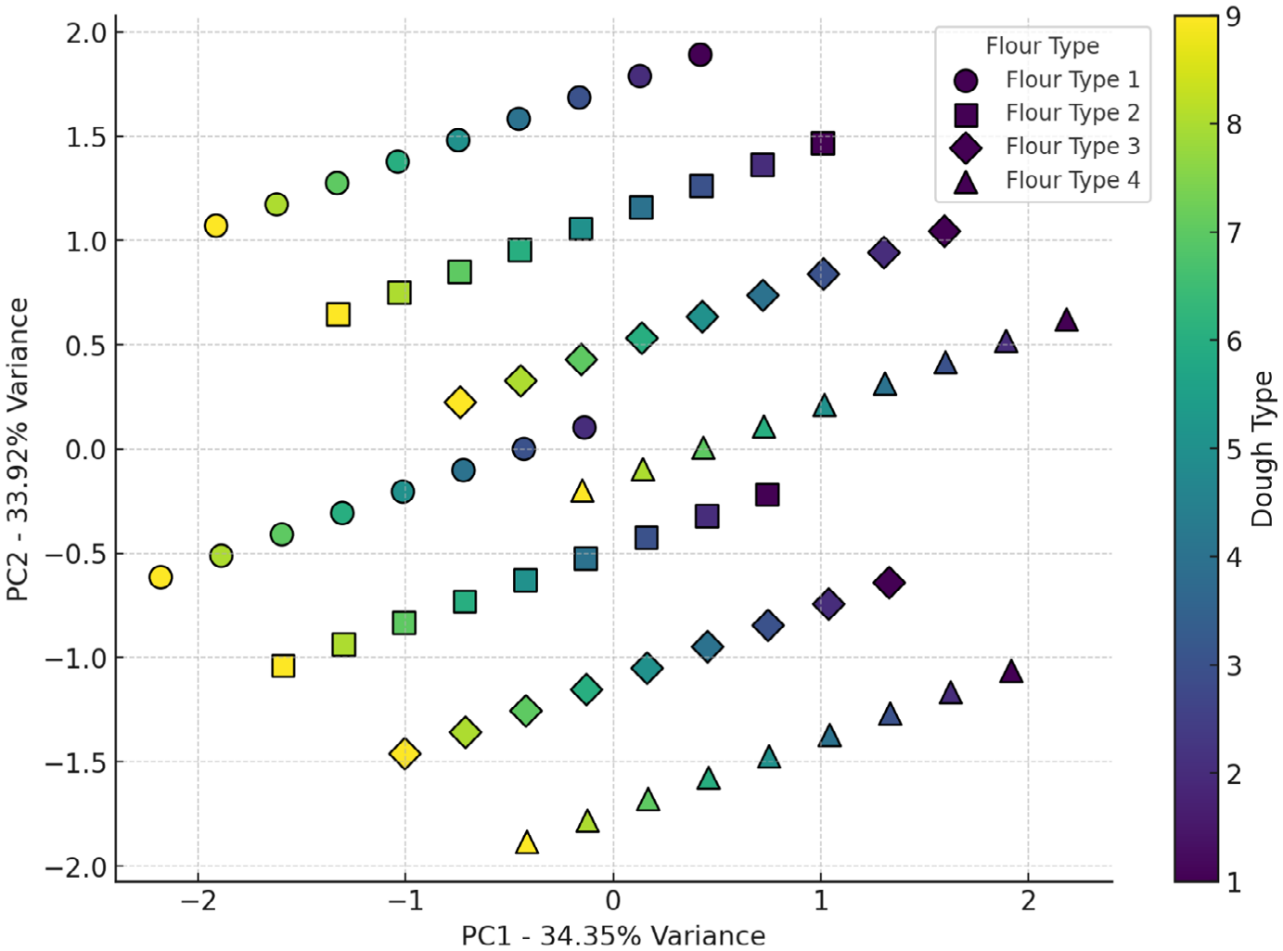
PCA analysis of the effect of different flour and sourdough combinations on bread pH. Flour type: 1 (wheat), 2 (5% oat), 3 (10% oat), 4 (15% oat); Sourdough type: 1 (wheat + *L. plantarum* ), 2 (oat + *L. plantarum* ), 3 (wheat + oat + *L. plantarum* ), 4 (wheat + *Sc. cerevisiae*), 5 (oat + *Sc. cerevisiae*), 6 (oat + wheat + *Sc. cerevisiae*), 7 ( *L. plantarum* + *Sc. cerevisiae* + wheat), 8 (oat + *L. plantarum* + *Sc. cerevisiae*), 9 (wheat + oat + *L. plantarum* + *Sc. cerevisiae*).

**FIGURE 4:**
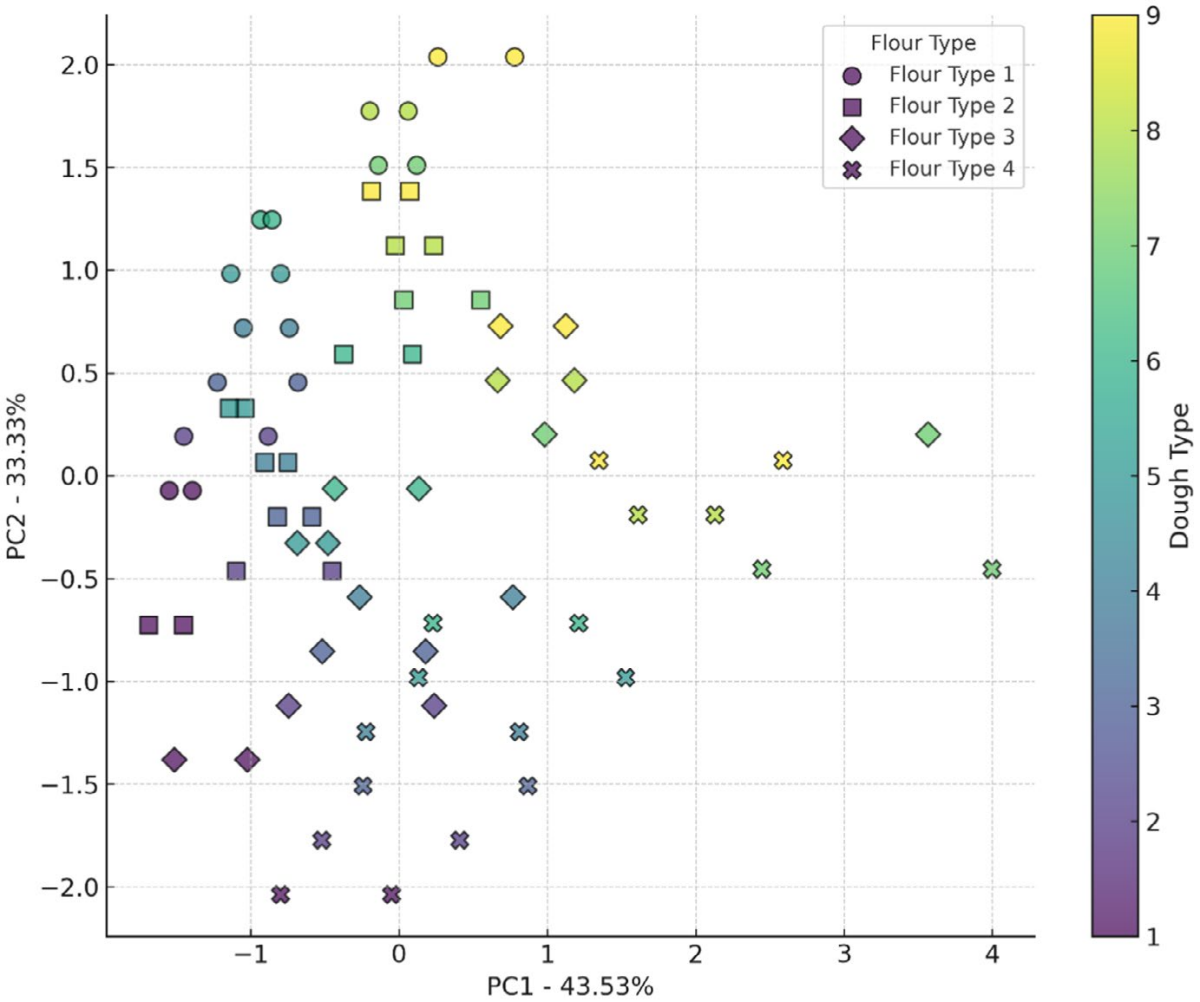
PCA analysis of the effect of different flour and sourdough combinations on bread a_w_. Flour type: 1 (wheat), 2 (5% oat), 3 (10% oat), 4 (15% oat); Sourdough type: 1 (wheat + *L. plantarum* ), 2 (oat + *L. plantarum* ), 3 (wheat + oat + *L. plantarum* ), 4 (wheat + *Sc. cerevisiae*), 5 (oat + *Sc. cerevisiae*), 6 (oat + wheat + *Sc. cerevisiae*), 7 ( *L. plantarum* + *Sc. cerevisiae* + wheat), 8 (oat + *L. plantarum* + *Sc. cerevisiae*), 9 (wheat + oat + *L. plantarum* + *Sc. cerevisiae*).

**FIGURE 5:**
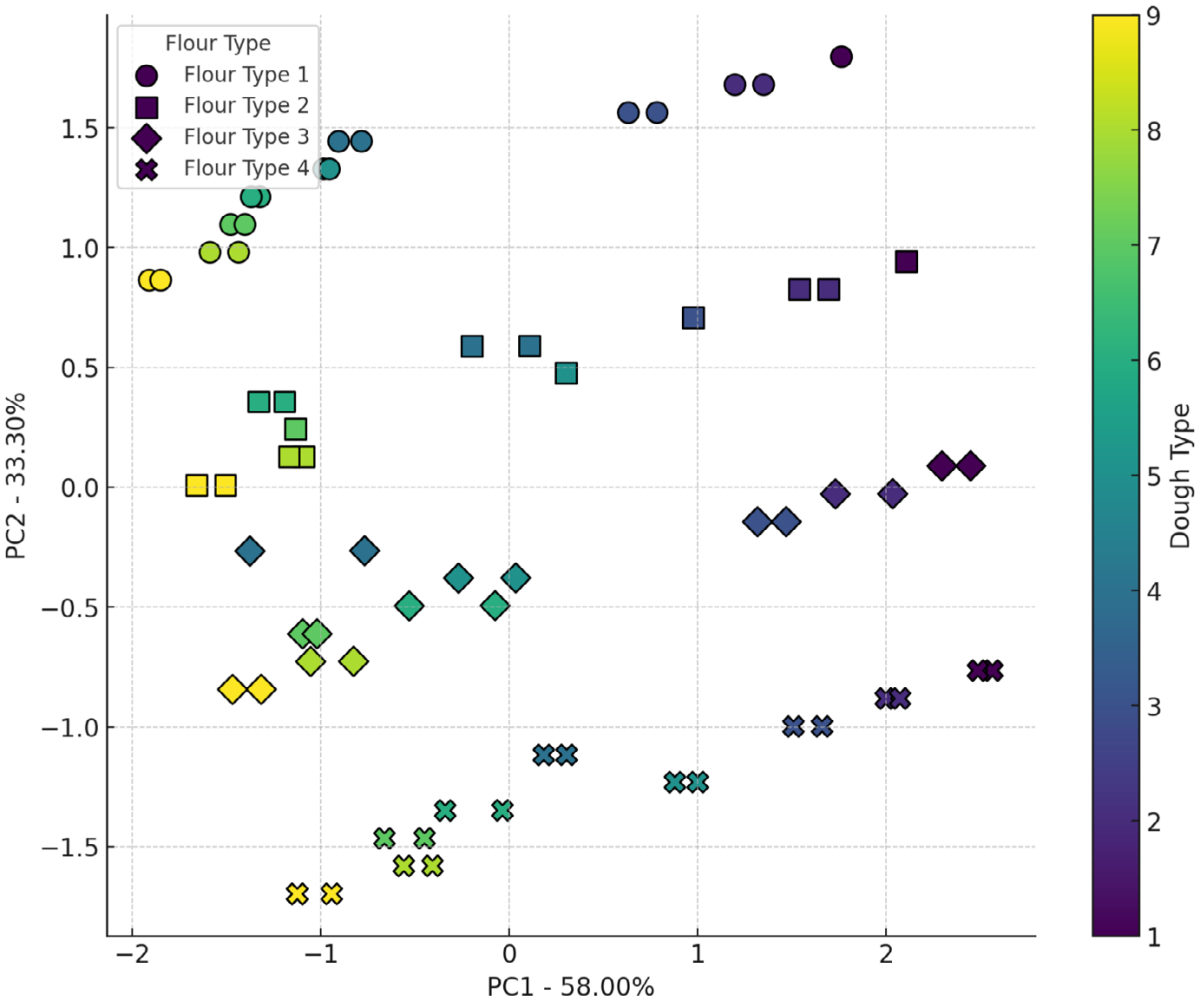
PCA analysis of the effect of different flour and sourdough combinations on bread Specific volume. Flour type: 1 (wheat), 2 (5% oat), 3 (10% oat), 4 (15% oat); Sourdough type: 1 (wheat + *L. plantarum* ), 2 (oat + *L. plantarum* ), 3 (wheat + oat + *L. plantarum* ), 4 (wheat + *Sc. cerevisiae*), 5 (oat + *Sc. cerevisiae*), 6 (oat + wheat + *Sc. cerevisiae*), 7 ( *L. plantarum* + *Sc. cerevisiae* + wheat), 8 (oat + *L. plantarum* + *Sc. cerevisiae*), 9 (wheat + oat + *L. plantarum* + *Sc. cerevisiae*).

**FIGURE 6:**
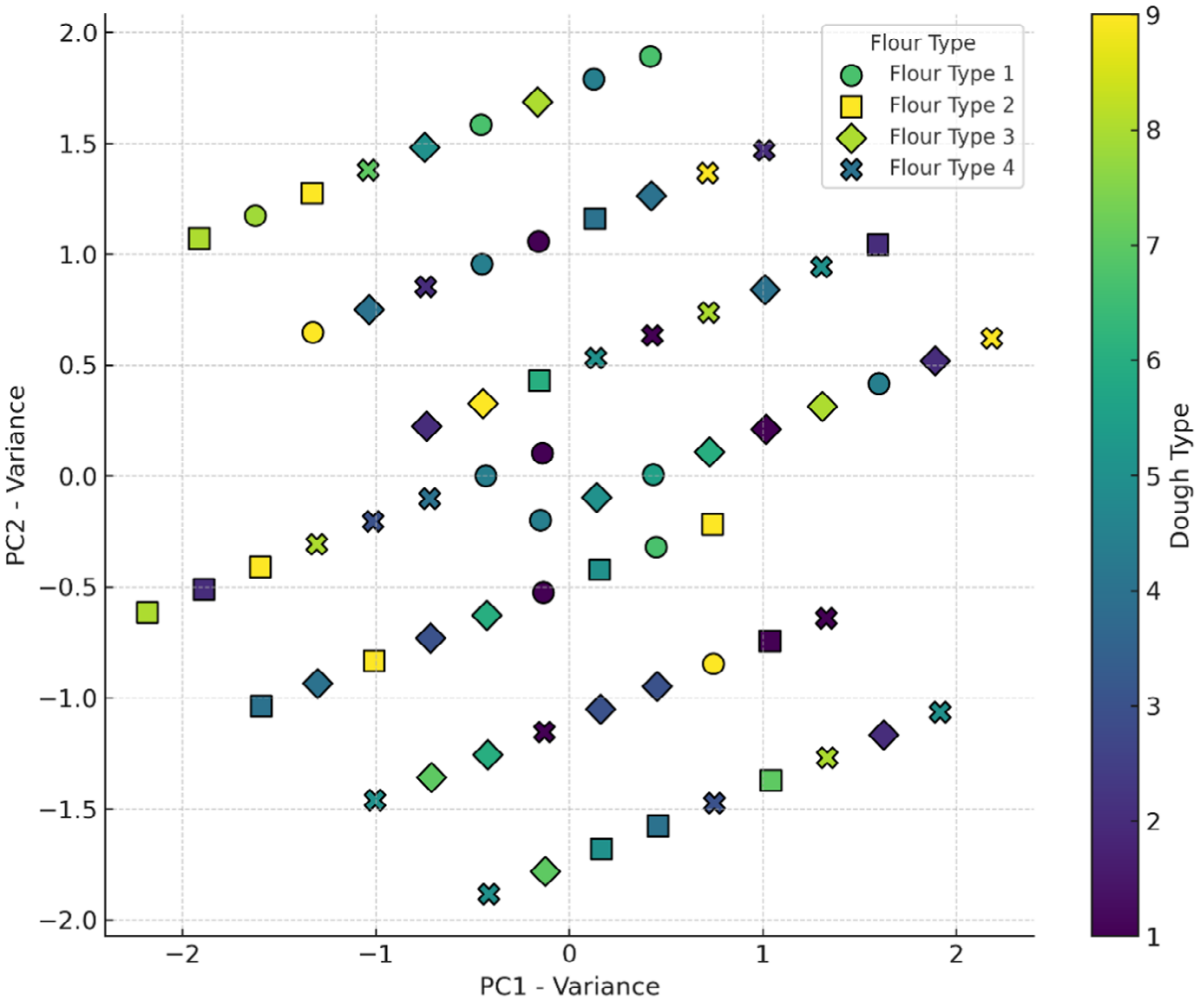
PCA analysis of the effect of different flour and sourdough combinations on bread fiber. Flour type: 1 (wheat), 2 (5% oat), 3 (10% oat), 4 (15% oat); Sourdough type: 1 (wheat + *L. plantarum* ), 2 (oat + *L. plantarum* ), 3 (wheat + oat + *L. plantarum* ), 4 (wheat + *Sc. cerevisiae*), 5 (oat + *Sc. cerevisiae*), 6 (oat + wheat + *Sc. cerevisiae*), 7 ( *L. plantarum* + *Sc. cerevisiae* + wheat), 8 (oat + *L. plantarum* + *Sc. cerevisiae*), 9 (wheat + oat + *L. plantarum* + *Sc. cerevisiae*).

**FIGURE 7:**
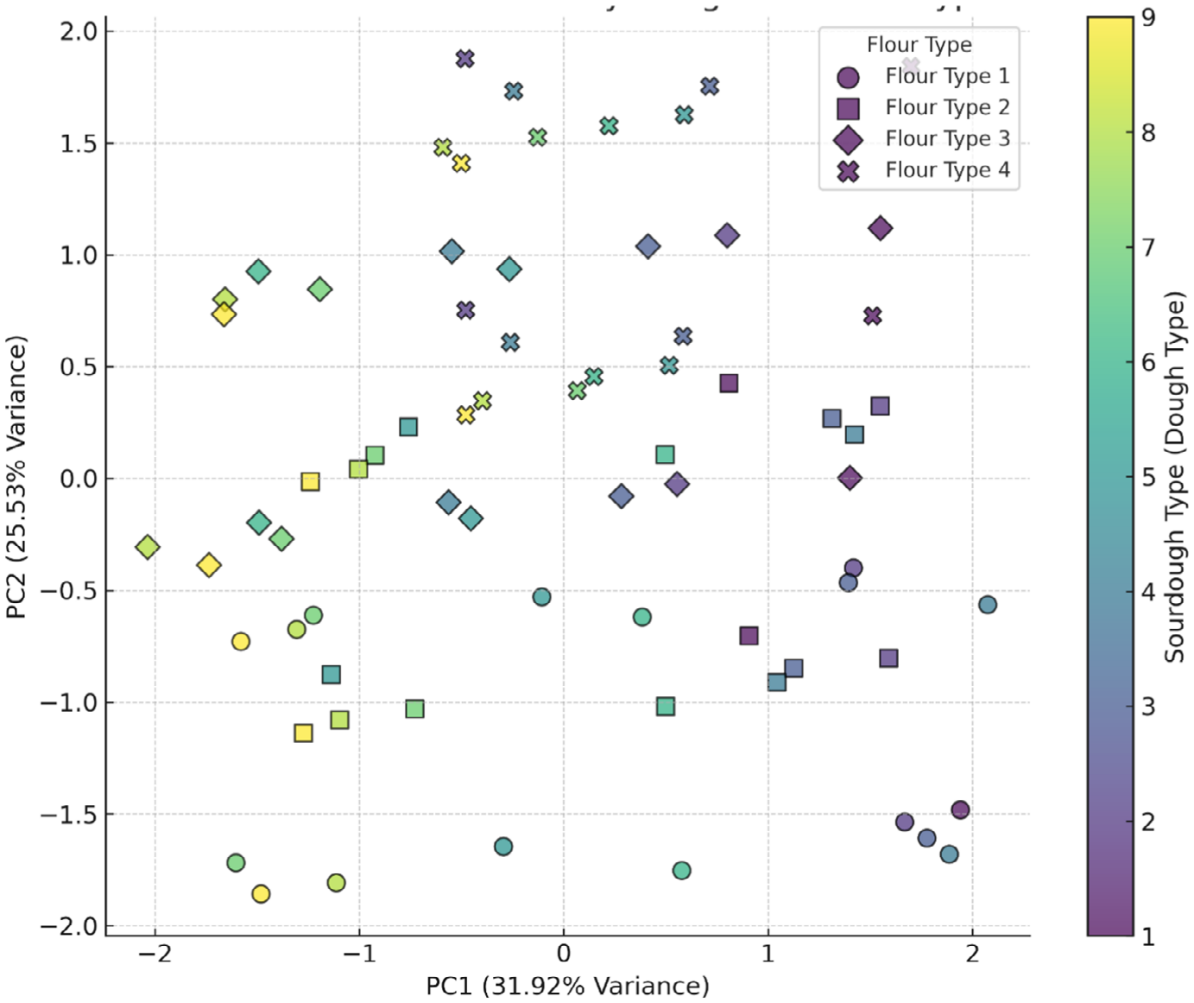
PCA analysis of the effect of different flour and sourdough combinations on bread protein. Flour type: 1 (wheat), 2 (5% oat), 3 (10% oat), 4 (15% oat); Sourdough type: 1 (wheat + *L. plantarum* ), 2 (oat + *L. plantarum* ), 3 (wheat + oat + *L. plantarum* ), 4 (wheat + *Sc. cerevisiae*), 5 (oat + *Sc. cerevisiae*), 6 (oat + wheat + *Sc. cerevisiae*), 7 ( *L. plantarum* + *Sc. cerevisiae* + wheat), 8 (oat + *L. plantarum* + *Sc. cerevisiae*), 9 (wheat + oat + *L. plantarum* + *Sc. cerevisiae*).

**FIGURE 8:**
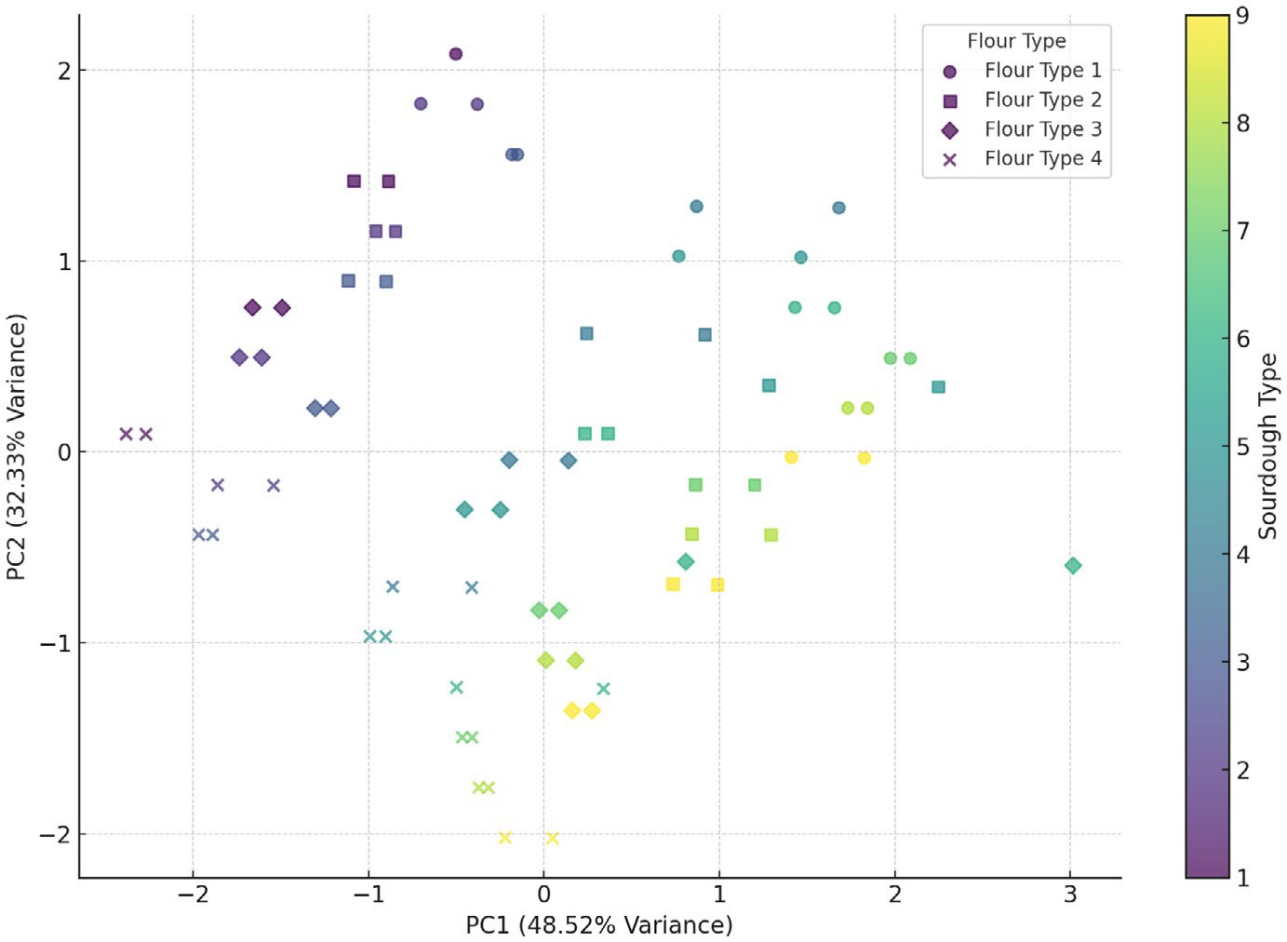
PCA analysis of the effect of different flour and sourdough combinations on bread Porosity. Flour type: 1 (wheat), 2 (5% oat), 3 (10% oat), 4 (15% oat); Sourdough type: 1 (wheat + *L. plantarum* ), 2 (oat + *L. plantarum* ), 3 (wheat + oat + *L. plantarum* ), 4 (wheat + *Sc. cerevisiae*), 5 (oat + *Sc. cerevisiae*), 6 (oat + wheat + *Sc. cerevisiae*), 7 ( *L. plantarum* + *Sc. cerevisiae* + wheat), 8 (oat + *L. plantarum* + *Sc. cerevisiae*), 9 (wheat + oat + *L. plantarum* + *Sc. cerevisiae*).

Values for Moisture, Enhancement in dough volume, pH, water activity (a_w_), specific volume, fiber, protein, and porosity are given in Figures 1, 2, 3, 4, 5, 6, 7, 8. The results of PCA analysis on the effect of sourdough and the percentage of wheat and oat flour in the bread formulation on moisture (Figure 1) showed that component 1 and component 2 explain approximately 68.27% of the total variance in the data. Wheat flour, used in various sourdough combinations, showed a mild effect on bread moisture, with less variability than other flours. Wheat flour + 5% oat flour significantly influenced moisture in certain doughs, creating similar moisture characteristics in bread. Wheat flour + 10% oat flour tended to decrease moisture, as indicated by its positioning in the lower chart regions. Wheat flour + 15% oat flour showed greater flexibility in adjusting moisture levels, making it suitable for diverse moisture properties. Wheat flour and wheat flour + 15% oat flour display broader distribution, suggesting less consistent moisture effects, while wheat flour + 5% oat flour and wheat flour + 10% oat flour cluster in specific areas, indicating more defined impacts on moisture. Sourdoughs in the upper and mid‐regions of the chart (codes 1 and 2) generally increased moisture, while those in the lower areas (codes 5, 7, 8, and 9) were more likely to reduce it. Mid‐level sourdoughs, like codes 3 and 4, seem balanced, and suitable for moderate moisture levels. Yeast fermentation enhances dough hydration by increasing the water‐holding capacity of the dough. As yeast cells consume sugars and produce metabolites, the dough becomes more extensible and elastic, allowing it to retain more water. This improved hydration contributes to a softer, moister crumb texture in the baked bread (Sluimer 2005). The results of Ogunsakin et al. (2015) showed that the highest amount of moisture and protein were observed in sorghum bread produced with baker's yeast (Ogunsakin et al. 2015).

Figure 2 shows two components captured around 93.97% of the total variance in the data. This high cumulative variance indicates that the plot effectively summarizes most of the variability in the dataset. The combination of wheat flour and wheat flour + 5% oat flour with sourdoughs (codes 8 and 9) appears to result in the greatest increase in dough volume. This combination seems to create optimal conditions for dough expansion. Although the fermentation time for doughs containing *Lb*. *plantarum* was significantly longer than that for doughs with *Sc*. *cerevisiae*, there was still no observed increase in volume for the samples with *Lb*. *plantarum*. This lack of volume increase could be attributed to the different metabolic pathways of *Lb*. *plantarum*, which primarily produce lactic acid rather than carbon dioxide. Unlike *Sc*. *cerevisiae*, which produces gases that expand the dough, *Lb*. *plantarum's* fermentation process does not contribute to the gas production necessary for volume enhancement. Furthermore, the acidification by lactic acid bacteria might weaken the gluten network, thereby preventing any significant increase in dough volume (Tebben, Shen, and Li 2018). In the PCA analysis of pH (Figure 3), the first and second components together explain 79.48% of the variance in pH data. The combination of wheat flour with sourdoughs (codes 7, 8, and 9) tended to increase pH, while wheat flour + 15% oat flour with sourdough 9 (wheat + *L. plantarum* ) decreased pH. This distribution highlighted the varying effects of flour and sourdough on bread pH, and the clustering of samples in the plot indicates that certain combinations have more distinct impacts on pH. The reason for this is that the fermentation activity of *Lb. plantarum* causes the production of organic acids such as lactic acid, which increases the acidity and decreases the pH in the dough (Șerban et al. 2021).

Figure 4 illustrates the interaction effects of four flour types and nine sourdough types on the water activity of bread. The X‐axis (PC1) explains 43.25% of the data variance, and the Y‐axis (PC2) explains 33.33%. Wheat flour and wheat flour + 5% oat flour are primarily located in the upper right of the plot, indicating a higher water activity. In contrast, wheat flour + 10% oat flour and wheat flour + 15% oat flour are positioned in the lower left, showing a lower water activity. Sourdough codes 6 to 9, especially with wheat flour and wheat flour + 5% oat flour, tend to increase water activity, while sourdough codes 1 to 5, especially with wheat flour + 10% oat flour and wheat flour + 15% oat flour, show a lower tendency for water activity. The presence of organic acids, such as lactic acid, binds with water molecules, reducing the amount of free water available in the dough. This reduction in free water lowers the water activity (Schefer, Oest, and Rohn 2021). The combined effects of lactic acid production by *Lb. plantarum*, gas formation by yeast, and water‐binding properties of oat components result in a bread structure with reduced water activity (Sun et al. 2020).

The X‐axis in Figure 5 (PC1) explains 58% of the data variance, and the Y‐axis (PC2) explains 33.30%, together covering 91.30% of the total variance. Wheat flour is scattered across the plot, mainly in negative PC1 regions, suggesting its effect on specific volume depending on the sourdough type. Wheat flour + 5% oat flour clusters centrally in positive PC1 regions, indicating a tendency for medium to high specific volume. Wheat flour + 10% oat flour and wheat flour + 15% oat flour are in the upper right, showing a positive influence on a specific volume. Sourdoughs (codes 7, 8, and 9), particularly with wheat flour + 10% oat flour and wheat flour + 15% oat flour, are associated with lower specific volumes, as reflected by negative values on PC1 and PC2. *Sc. cerevisiae* is highly efficient in fermenting sugars and generating gas rapidly under optimal conditions. This gas is trapped in the dough, causing it to rise and expand during proofing and baking. The abundant production of carbon dioxide by yeast results in increased gas retention within the dough, leading to a higher specific volume (Vučurović et al. 2022). Findings by Hu et al. (2022) demonstrated that breads made solely with *Sc. cerevisiae* exhibited a greater specific volume in comparison to breads made with both *Sc. cerevisiae* and *Lb. plantarum* (Hu et al. 2022).

It can be seen from the fiber result (Figure 6) that the wheat flour is widely dispersed, suggesting flexibility with various sourdoughs, while wheat flour + 5% oat flour appears centrally, indicating a balanced effect. Wheat flour + 10% oat flour and wheat flour + 15% oat flour cluster in the upper regions, suggesting a positive influence on fiber content. Sourdoughs (codes 7, 8, and 9) showed a consistent impact and tended to cluster in specific areas, while sourdoughs (codes 1 to 3) exhibited more variation, interacting differently with various flours. Overall, breads made with wheat flour + 10% oat flour and wheat flour + 15% oat flour combined with sourdoughs (codes 7, 8, and 9) tended to show higher fiber content. The fiber content of these breads ranged from 0.39% to 0.575%. *Lb. plantarum*, as a lactic acid bacterium used in sourdough fermentation, can contribute to fiber retention in the bread. During fermentation, *Lb. plantarum* interacts with the oat components, potentially enhancing the retention of oat fiber in the bread matrix. *Lb. plantarum* has the ability to synthesize exopolysaccharides like glucan, thereby enhancing the dietary fiber content of the product (Akamine, Mansoldo, and Vermelho 2023). In contrast, *Sc. cerevisiae* primarily acts as a leavening agent in bread making and does not contribute significantly to fiber retention or modification. Yeast fermentation focuses on sugar metabolism and gas production rather than fiber interaction. As a result, bread produced with wheat and *Sc. cerevisiae* may have a lower overall fiber content compared to bread containing oats and *Lb. plantarum* (Batt and Tortorello 2014).

Analysis of protein results (Figure 7) indicated that the combination of wheat flour + 10% oat flour and wheat flour + 15% oat flour with sourdough (codes 1 to 5) has the greatest effect on increasing the protein content in bread. In contrast, the combination of wheat flour + 10% oat flour and wheat flour + 15% oat flour with sourdough (codes 6 to 9) resulted in lower protein content. This analysis aligns with the distribution of points in the PCA plot, highlighting the differential impact of flour and sourdough combinations on the protein content in bread. Wheat flour is naturally rich in proteins, particularly gluten‐forming proteins like glutenin and gliadin (Kartseva et al. 2023). While yeast primarily metabolizes carbohydrates, it also contributes to the breakdown and modification of proteins in the dough. This can lead to an increase in the availability and concentration of certain proteins in the final bread product (Fu et al. 2023). The PCA analysis of porosity (Figure 8) showed that the first component (*x*‐axis) explains approximately 48.52% of the variance, and the second component (*y*‐axis) explains about 32.33% of the variance. Together, these two components cover around 80.85% of the total variance in the data. Wheat flour and wheat flour + 5% oat flour generally increased porosity, while wheat flour + 15% oat flour resulted in the lowest porosity. Sourdoughs (8 and 9) contributed to increased porosity, whereas sourdoughs (codes 1 and 2) helped reduce porosity.

### 3.2 TPA Analysis

TPA parameters revealed that the textural properties of bread were significantly affected (*p* < 0.05) by sourdough (Table 4). Although the fermentation time for doughs containing *Lb*. *plantarum* was significantly longer than that for those with *Sc*. *cerevisiae*, the samples with *Lb*. *plantarum* still exhibited different textural properties, such as increased hardness and cohesiveness. This could be due to the distinct metabolic activities of *Lb*. *plantarum*, which primarily produces lactic acid. The extended fermentation time combined with the lactic acid production may have led to the strengthening of the gluten network, resulting in firmer and more cohesive dough. However, without the gas production associated with yeast fermentation, these changes in texture did not correspond to an increase in dough volume. During fermentation, yeast influences gluten development by releasing enzymes that break down proteins into smaller peptides. Well‐developed gluten contributes to bread texture, providing elasticity and a softer crumb. In breads without yeast, the absence of gas production and gluten development can result in a denser and harder texture (Gänzle 2014). *Lb. plantarum* produces lactic acid during fermentation, which lowers the pH of the dough. The increased acidity can affect the protein structure of the bread, including gluten, leading to changes in texture. Acid‐induced modifications in gluten can contribute to increased stickiness in the bread (Gänzle and Salovaara 2019). Breads lacking yeast and containing *Lb. plantarum* bacteria, whether with wheat, oat, or both, showed the lowest levels of springiness, while samples containing yeast and *Lb. plantarum* manifested the highest levels of springiness. Bread produced with *Lb. plantarum* and without yeast may exhibit weaker gluten development due to differences in fermentation dynamics and acidification. Weaker gluten structure can lead to reduced springiness in the final bread (Gobbetti et al. 2014).

**TABLE 4:** TPA analysis of Bread Produced using different formulations.

Sample code	Hardness N	Chewiness N	Cohesiveness	Springiness (cm^3^/g)
W100/W—*Lb*	6142.5 ± 3.53^h^	564.5 ± 6.364^f^	0.609 ± 0.013^cd^	7.4 ± 0.141^m^
W100/O—*Lb*	6100^h^	605.5 ± 6.364^b^	0.696 ± 0.006^a^	7.45 ± 0.071^m^
W100/W‐O—*Lb*	6711 ± 15.556^d^	544 ± 5.657^g^	0.696 ± 0.006^a^	7.05 ± 0.071^n^
W100/W—*Sc*	2405 ± 7.701^q^	266.8 ± 2.546^p^	0.405 ± 0.007^h^	17.25 ± 0.354^c^
W100/O—*Sc*	2006 ± 8.485^s^	172.6 ± 3.677^w^	0.426 ± 0.008^g^	18.1 ± 0.141^a^
W100/W‐O—*Sc*	2520.5 ± 28.99^p^	216.35 ± 1.909^s^	0.45 ± 0.013^fg^	16.35 ± 0.212^e^
W100/W—*Lb*—*Sc*	2900 ± 141.421^n^	405 ± 7.071^j^	0.565 ± 0.021^d^	7.9 ± 0.141^lm^
W100/O—*Lb*—*Sc*	2600 ± 141.41^o^	462.5 ± 3.536^h^	0.475 ± 0.035^f^	9.3 ± 0.424^k^
W100/W‐O—*Lb*—*Sc*	2352 ± 2.828^q^	201.5 ± 2.121^t^	0.488 ± 0.018^f^	16.05 ± 0.071^f^
W95/O5/W—*Lb*	6860 ± 14.141^c^	605 ± 7.071^b^	0.551 ± 0.071^de^	7.55 ± 0.071^m^
W95/O5/O—*Lb*	6557.500 ± 10.61^e^	595 ± 7.071^cd^	0.549 ± 0.069^de^	6.95 ± 0.071^n^
W95/O5/W‐O—*Lb*	6838.5 ± 12.021^c^	597.5 ± 4.95^c^	0.616 ± 0.023^c^	7.1 ± 0.141^n^
W95/O5/W—*Sc*	2453.5 ± 4.95^q^	332.55 ± 3.606^m^	0.403 ± 0.004^h^	17.6 ± 0.141^b^
W95/O5/O—*Sc*	2449 ± 12.728^q^	181.65 ± 2.333^v^	0.382 ± 0.016^i^	16.15 ± 0.212^e^
W95/O5/W‐O—*Sc*	2156^r^	242.95 ± 2.899^r^	0.413 ± 0.018^gh^	13.725 ± 0.035^i^
W95/O5/W—*Lb*—*Sc*	5550 ± 70.711^k^	282.5 ± 3.536^o^	0.525 ± 0.035^e^	9.2 ± 0.283^k^
W95/O5/O—*Lb*—*Sc*	4900 ± 141.421^m^	295 ± 7.071^n^	0.465 ± 0.021^f^	10.25 ± 0.354^j^
W95/O5/W‐O—*Lb*—*Sc*	1960 ± 7.071^s^	181.4 ± 1.98^v^	0.387 ± 0.009^i^	16.4 ± 0.141^e^
W90/O10/W—*Lb*	6215.5 ± 21.92^g^	573.5 ± 4.95^e^	0.609 ± 0.013^cd^	6.45 ± 0.071^o^
W90/O10/O—*Lb*	6306 ± 8.485^f^	583.5 ± 4.95^d^	0.579 ± 0.012^de^	7.45 ± 0.071^m^
W90/O10/W‐O—*Lb*	6153 ± 4.243^h^	600 ± 2.828^c^	0.595 ± 0.006^d^	7.45 ± 0.071^m^
W90/O10/W—*Sc*	3000.5 ± 0.707^n^	361.95 ± 2.758^l^	0.427 ± 0.009^g^	15.55 ± 0.071^f^
W90/O10/O—*Sc*	2163 ± 18.385^r^	253 ± 4.243^q^	0.45 ± 0.001^fg^	13.45 ± 0.071^i^
W90/O10/W‐O—*Sc*	2022.5 ± 3.536^r^	156.35 ± 3.748^x^	0.361 ± 0.015^j^	14.1 ± 0.141^h^
W90/O10/W—*Lb*—*Sc*	5450 ± 70.711^k^	384.5 ± 21.92^k^	0.475 ± 0.007^f^	8.25 ± 0.354^l^
W90/O10/O—*Lb*—*Sc*	4850 ± 212.132^m^	345 ± 7.071^l^	0.43 ± 0.014^g^	9.25 ± 0.354^k^
W90/O10/W‐O—*Lb*—*Sc*	2100.5 ± 0.707^r^	192.65 ± 3.748^u^	0.394 ± 0.005^i^	15.4 ± 0.141^f^
W85/O15/W—*Lb*	5983.5 ± 4.95^i^	571 ± 1.414^e^	0.574 ± 0.019^de^	6.45 ± 0.071^o^
W85/O15/O—*Lb*	6983.5 ± 4.95^b^	616 ± 1.414^a^	0.631 ± 0.015^b^	6.45 ± 0.0771^o^
W85/O15/W‐O—*Lb*	7842.5 ± 3.536^a^	600 ± 1.414^c^	0.606 ± 0.008^cd^	7.1 ± 0.141^n^
W85/O15/W—*Sc*	2993.5 ± 7.778^n^	290.85 ± 1.202^n^	0.445 ± 0.007^fg^	14.05 ± 0.071^h^
W85/O15/O—*Sc*	3101 ± 1.414^n^	336.85 ± 2.616^m^	0.417 ± 0.009^h^	15.450 ± 0.071^f^
W85/O15/W‐O—*Sc*	2193 ± 4.243^r^	171.35 ± 0.919^w^	0.398 ± 0.003^i^	14.550 ± 0.071^g^
W85/O15/W—*Lb*—*Sc*	5600 ± 141.421^j^	410 ± 14.142^j^	0.51 ± 0.014^e^	8.250 ± 0.354^l^
W85/O15/O—*Lb*—*Sc*	5250 ± 212.132^l^	432.5 ± 3.536^i^	0.435 ± 0.049^g^	9.15 ± 0.212^k^
W85/O15/W‐O—*Lb*—*Sc*	1982.5 ± 3.536^s^	198.6 ± 1.98^u^	0.388 ± 0.011^i^	17.05 ± 0.071^d^

*Note:* Different letters within column indicates significant different between means (*p* < 0.05).

### 3.3 Color Measurement

The color of the bread crust and crumb is reported in Table 5. Sourdough without the addition of *Sc. cerevisiae* had a notable impact on the colorimetric data, resulting in a lightening effect by increasing the L* value in crust from 72.35 to 72.94 with no significant difference (*p* < 0.05). This trend was also observed in the bread crumb. Bread produced with both *Lb. plantarum* and *Sc. cerevisiae* may undergo more extensive browning reactions due to the combined effects of lactic acid fermentation and yeast fermentation, resulting in darker crust and crumb color and lower L* values compared to bread produced solely with *Lb. plantarum* (Ferraz et al. 2021). The results of Torrieri et al. (2014) did not match our findings; they showed that adding a higher dose of sourdough without *Sc. cerevisiae* led to an increase in the L* index in bread (Torrieri et al. 2014). The sourdough used in bread production caused the highest amount of a*, b* and Δ*E* indices in the crust, while breads without yeast had the lowest values. The highest a* and b* values observed in bread produced with oat, wheat, *Lb. plantarum*, and *Sc. cerevisiae* yeast. These values are attributed to the synergistic effects of yeast and lactic acid fermentation, the Maillard reaction, flour composition, and baking conditions. These factors contribute to the development of reddish‐brown and yellow hues in the bread crust, as measured by the HunterLab colorimetry system (Torrieri et al. 2014). The lowest a*, b* and Δ*E* indices were displayed in the bread crumb containing oat and *Sc. cerevisiae*.

**TABLE 5:** Color analysis of bread produced using Hunterlab.

Treatment	Crust				Crumb			
	L*	a*	b*	Δ*E*	L*	a*	b*	Δ*E*
T3	72.5 ± 0.71^a^	2.45 ± 0.07^c^	23.05 ± 0.07^f^	0.12 ± 0.01^e^	72.65^a^	0.29 ± 0.01^e^	22.4 ± 0.14^a^	0.01^h^
T4	61.24 ± 0.08^b^	10.13 ± 0.05^b^	30.51 ± 0.05^c^	0.44 ± 0.01^d^	69 ± 0.04^b^	−0.08 ± 0.01^d^	22.7 ± 0.05^a^	0^g^
T5	59.5 ± 0.71^bc^	11.3 ± 0.14^a^	27.7 ± 0.28^d^	0.55 ± 0.07^b^	72.95 ± 0.07^a^	0.95 ± 0.07^f^	22.2 ± 0.28^b^	−0.05 ± 0.001^b^
T6	57.5 ± 0.71^d^	11.25 ± 0.35^a^	35.15 ± 0.21^a^	0.55 ± 0.07^b^	70.75 ± 0.35^b^	−0.45 ± 0.07^c^	22.15 ± 0.21^b^	−0.01^f^
T9	56.75 ± 0.35^de^	11.25 ± 0.35^a^	25.95 ± 0.07^e^	0.53 ± 0.04^b^	69.135 ± 0.21^b^	−0.73 ± 0.04^b^	21.25 ± 0.35^c^	−0.04^c^
T12	72.95 ± 0.35^a^	2.46 ± 0.26^c^	23.25 ± 0.56^f^	0.13 ± 0.01^e^	72.9 ± 0.17^a^	0.28 ± 0.01^e^	22.47 ± 0.09^a^	0.02^i^
T13	60.5 ± 0.71^b^	10^b^	30.2 ± 0.28^c^	0.42 ± 0.03^d^	68 ± 0.71^c^	−0.07 ± 0.01^d^	22.6 ± 0.14^a^	0^g^
T14	60.09 ± 0.17^b^	11.11 ± 0.16^a^	28.14 ± 0.5^d^	0.51^c^	72.92 ± 0.12^a^	−1 ± 0.01^a^	22.46 ± 0.06^a^	−0.05^b^
T15	56.75 ± 0.35^de^	11.15 ± 0.21^a^	34.5 ± 0.71^b^	0.5^c^	70.5 ± 0.71^b^	−0.39 ± 0.02^c^	21.9 ± 0.14^b^	−0.02^e^
T18	56.5 ± 0.71^de^	11.45 ± 0.07^a^	25.9 ± 0.14^e^	0.58 ± 0.04^a^	68.5 ± 0.14^bc^	−0.8^b^	21.1 ± 0.14^c^	−0.04^c^
T21	72.5 ± 0.71^a^	2.55 ± 0.07^c^	23.25 ± 0.35^f^	0.13^e^	73.35 ± 0.71^a^	0.28 ± 0.01^e^	22.55 ± 0.07^a^	0.02^i^
T22	59 ± 1.41^c^	9.75 ± 0.35^b^	30.5 ± 0.71^c^	0.45 ± 0.07^d^	67 ± 0.7^c^	−0.33 ± 0.39^c^	22.5 ± 0.71^a^	0^g^
T23	60.5 ± 0.71^b^	11.25 ± 0.35^a^	27.7 ± 0.28^d^	0.58 ± 0.04^a^	73.1 ± 0.14^a^	−0.98 ± 0.04^a^	22.65 ± 0.07^a^	−0.06 ± 0.001^a^
T24	57.11 ± 0.54^d^	11.22 ± 0.08^a^	35.35 ± 1.06^a^	0.44 ± 0.01^d^	69.79 ± 0.52^b^	−0.36 ± 0.02^c^	21.95 ± 0.23^b^	−0.02^e^
T27	55.75 ± 0.35^e^	11.7 ± 0.14^a^	26.1 ± 0.14^e^	0.59 ± 0.14^a^	69.5 ± 0.21^b^	−0.75 ± 0.07^b^	20.8 ± 0.28^d^	−0.04^c^
T30	72.35 ± 0.49^a^	2.63 ± 0.04^c^	22.75 ± 0.35^fg^	0.14^e^	73.54 ± 0.57^a^	0.28 ± 0.01^e^	22.65 ± 0.21^a^	0.01^h^
T31	59^c^	10.10 ± 0.14^b^	30.25 ± 1.06^c^	0.43 ± 0.04^d^	67 ± 0.21^c^	−0.05^d^	22.75 ± 0.21^a^	0^g^
T32	60.4 ± 0.57^b^	10.95 ± 0.07^b^	28.1 ± 0.14^d^	0.54 ± 0.06^b^	72.5 ± 0.71^a^	−0.97 ± 0.03^a^	12.5 ± 0.15^e^	−0.06 ± 0.001^a^
T33	56.5 ± 0.71^de^	11.15 ± 0.21^a^	34.5 ± 0.71^b^	0.43 ± 0.04^d^	69.5 ± 0.7^b^	−0.38 ± 0.04^c^	21.8 ± 0.28^b^	−0.03 ± 0.001^d^
T36	56.39 ± 0.76^de^	11.54 ± 0.08^a^	26.4 ± 0.71^e^	0.57 ± 0.01^a^	70.15 ± 0.18^b^	−0.78 ± 0.01^b^	20.5 ± 0.3^d^	−0.04^c^

*Note:* Different letters within column indicates significant different between means (*p* < 0.05).

### 3.4 Bread Microstructure by SEM

**FIGURE 9:**
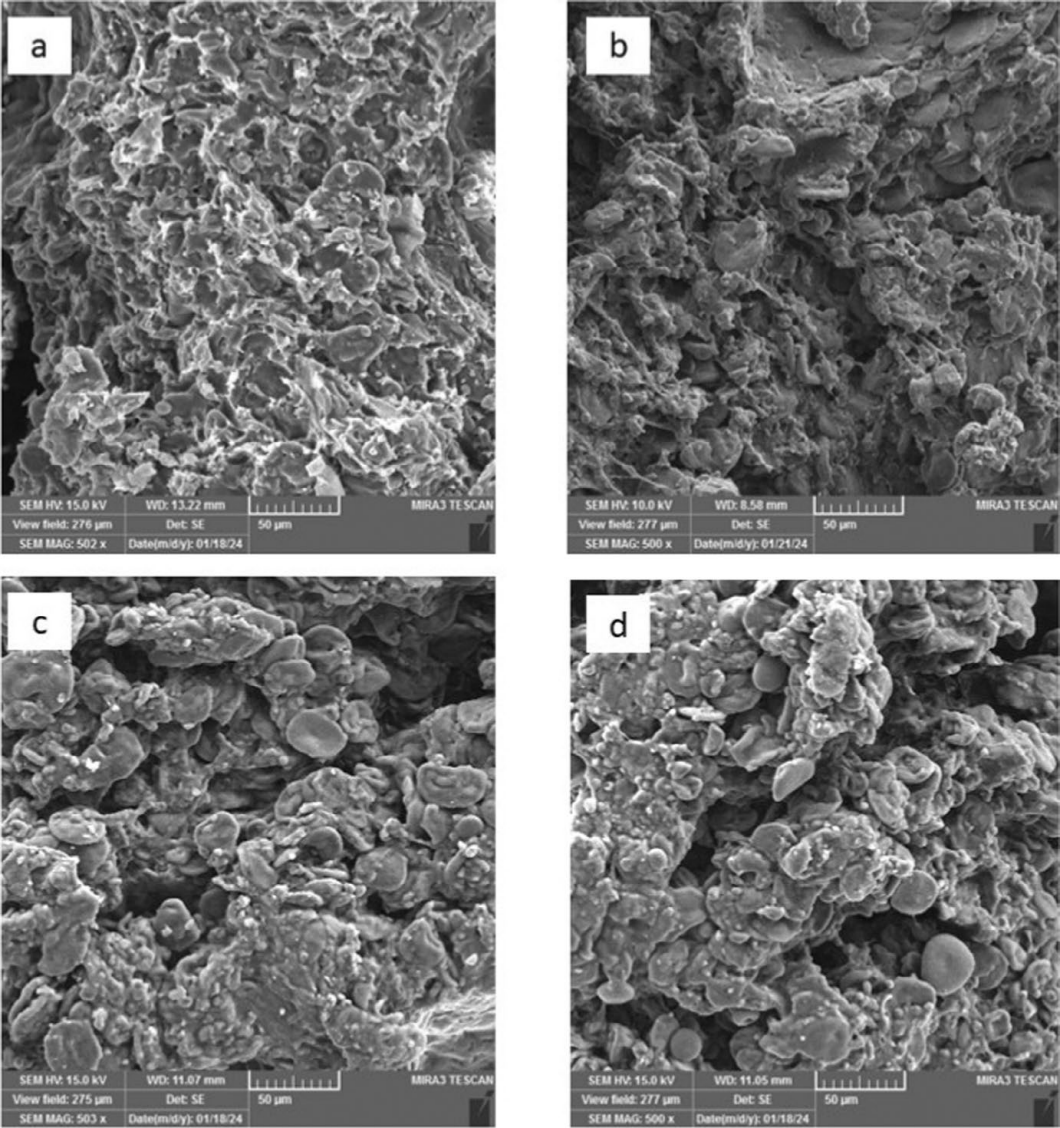
Microstructure of baked bread with different oat flour ratios and sourdough. (a) without oat flour (T9), (b) 5% oat flour (T18) (c) 10% oat flour (T27) d) 15% oat flour (T36).

Figure 9 shows the microstructure of breads baked with sourdough (wheat, oat, *Lb. plantarum*, and *Sc. cerevisiae*) in different ratios of oat flour. SEM analysis revealed that reducing the amount of wheat flour in a bread formulation can lead to increased porosity. The porosity data confirmed this observation, with samples formulated with 10% and 15% oat flour exhibiting larger pores. Substituting wheat flour with oat flour, which has lower gluten content and different protein compositions, also affects the bread's porosity. This substitution results in a weaker gluten network, contributing to increased porosity (Gajula 2017). Our findings are consistent with those of Korus et al. (2020) who observed that adding citrus fiber to bread increased porosity and average particle size (Korus et al. 2020). However, the results of Al‐Attabi et al. (2017) contradicted our outcomes. They reported that increasing the proportion of oat in bread from 10% upward resulted in a reduction in bread porosity (Al‐Attabi et al. 2017).

### 3.5 Sensory Evaluation

**FIGURE 10:**
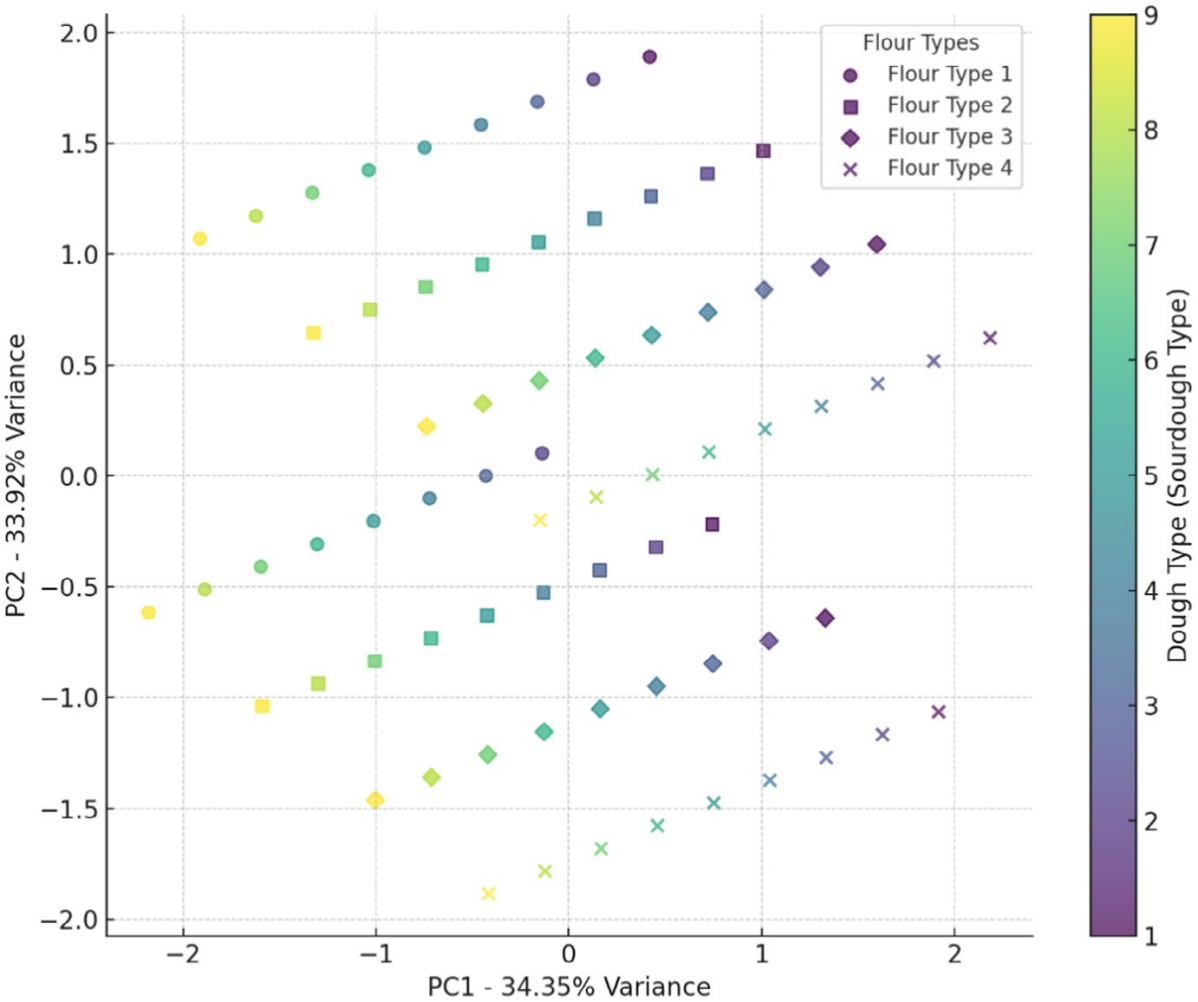
PCA analysis of the effect of different flour and sourdough combinations on bread Taste. Flour type: 1 (wheat), 2 (wheat + 5% oat), 3 (wheat + 10% oat), 4 (wheat + 15% oat); Sourdough type: 1 (wheat + *L. plantarum* ), 2 (oat + *L. plantarum* ), 3 (wheat + oat + *L. plantarum* ), 4 (wheat + *Sc. cerevisiae*), 5 (oat + *Sc. cerevisiae*), 6 (oat + wheat + *Sc. cerevisiae*), 7 ( *L. plantarum* + *Sc. cerevisiae* + wheat), 8 (oat + *L. plantarum* + *Sc. cerevisiae*), 9 (wheat + oat + *L. plantarum* + *Sc. cerevisiae*).

**FIGURE 11:**
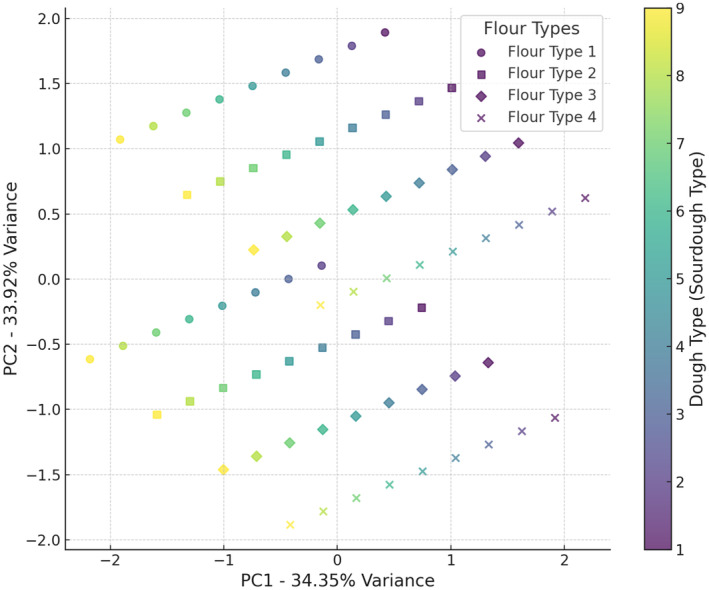
PCA analysis of the effect of different flour and sourdough combinations on bread Odor. Flour type: 1 (wheat), 2 (wheat + 5% oat), 3 (wheat + 10% oat), 4 (wheat + 15% oat); Sourdough type: 1 (wheat + *L. plantarum* ), 2 (oat + *L. plantarum* ), 3 (wheat + oat + *L. plantarum* ), 4 (wheat + *Sc. cerevisiae*), 5 (oat + *Sc. cerevisiae*), 6 (oat + wheat + *Sc. cerevisiae*), 7 ( *L. plantarum* + *Sc. cerevisiae* + wheat), 8 (oat + *L. plantarum* + *Sc. cerevisiae*), 9 (wheat + oat + *L. plantarum* + *Sc. cerevisiae*).

**FIGURE 12:**
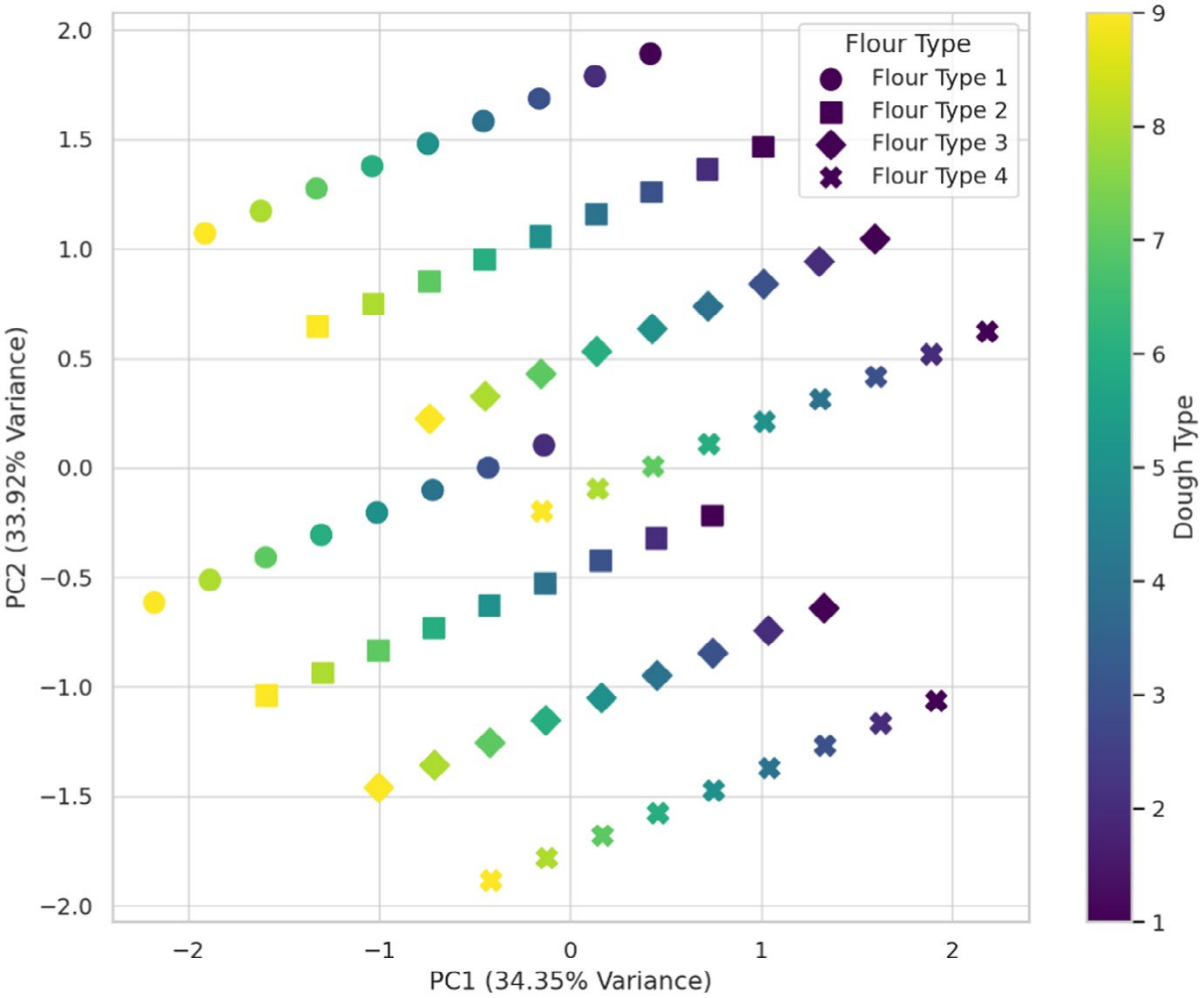
PCA analysis of the effect of different flour and sourdough combinations on bread Appearance. Flour type: 1 (wheat), 2 (wheat + 5% oat), 3 (wheat + 10% oat), 4 (wheat + 15% oat); Sourdough type: 1 (wheat + *L. plantarum* ), 2 (oat + *L. plantarum* ), 3 (wheat + oat + *L. plantarum* ), 4 (wheat + *Sc. cerevisiae*), 5 (oat + *Sc. cerevisiae*), 6 (oat + wheat + *Sc. cerevisiae*), 7 ( *L. plantarum* + *Sc. cerevisiae* + wheat), 8 (oat + *L. plantarum* + *Sc. cerevisiae*), 9 (wheat + oat + *L. plantarum* + *Sc. cerevisiae*).

**FIGURE 13:**
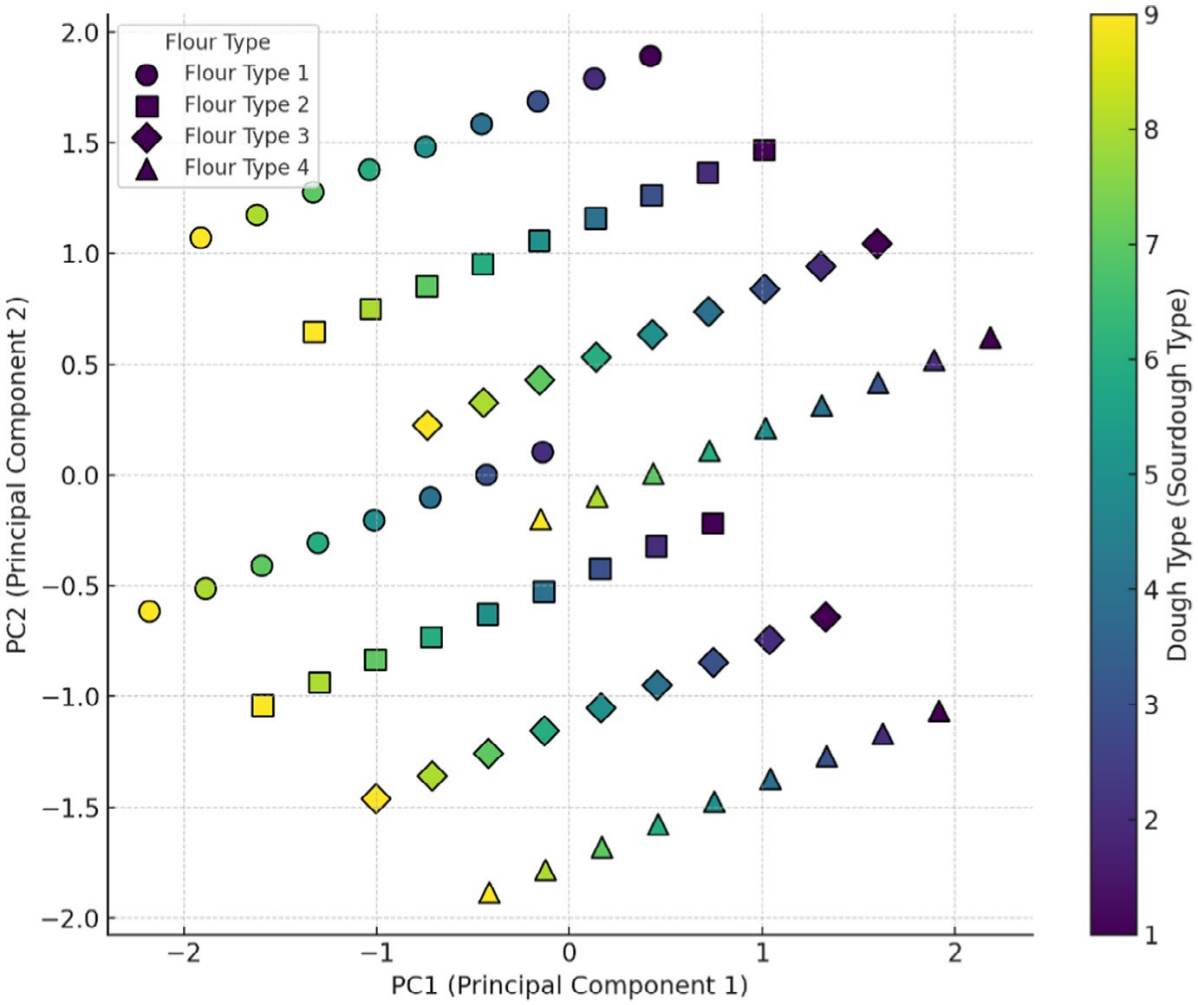
PCA analysis of the effect of different flour and sourdough combinations on bread Texture. Flour type: 1 (wheat), 2 (wheat + 5% oat), 3 (wheat + 10% oat), 4 (wheat + 15% oat); Sourdough type: 1 (wheat + *L. plantarum* ), 2 (oat + *L. plantarum* ), 3 (wheat + oat + *L. plantarum* ), 4 (wheat + *Sc. cerevisiae*), 5 (oat + *Sc. cerevisiae*), 6 (oat + wheat + *Sc. cerevisiae*), 7 ( *L. plantarum* + *Sc. cerevisiae* + wheat), 8 (oat + *L. plantarum* + *Sc. cerevisiae*), 9 (wheat + oat + *L. plantarum* + *Sc. cerevisiae*).

**FIGURE 14:**
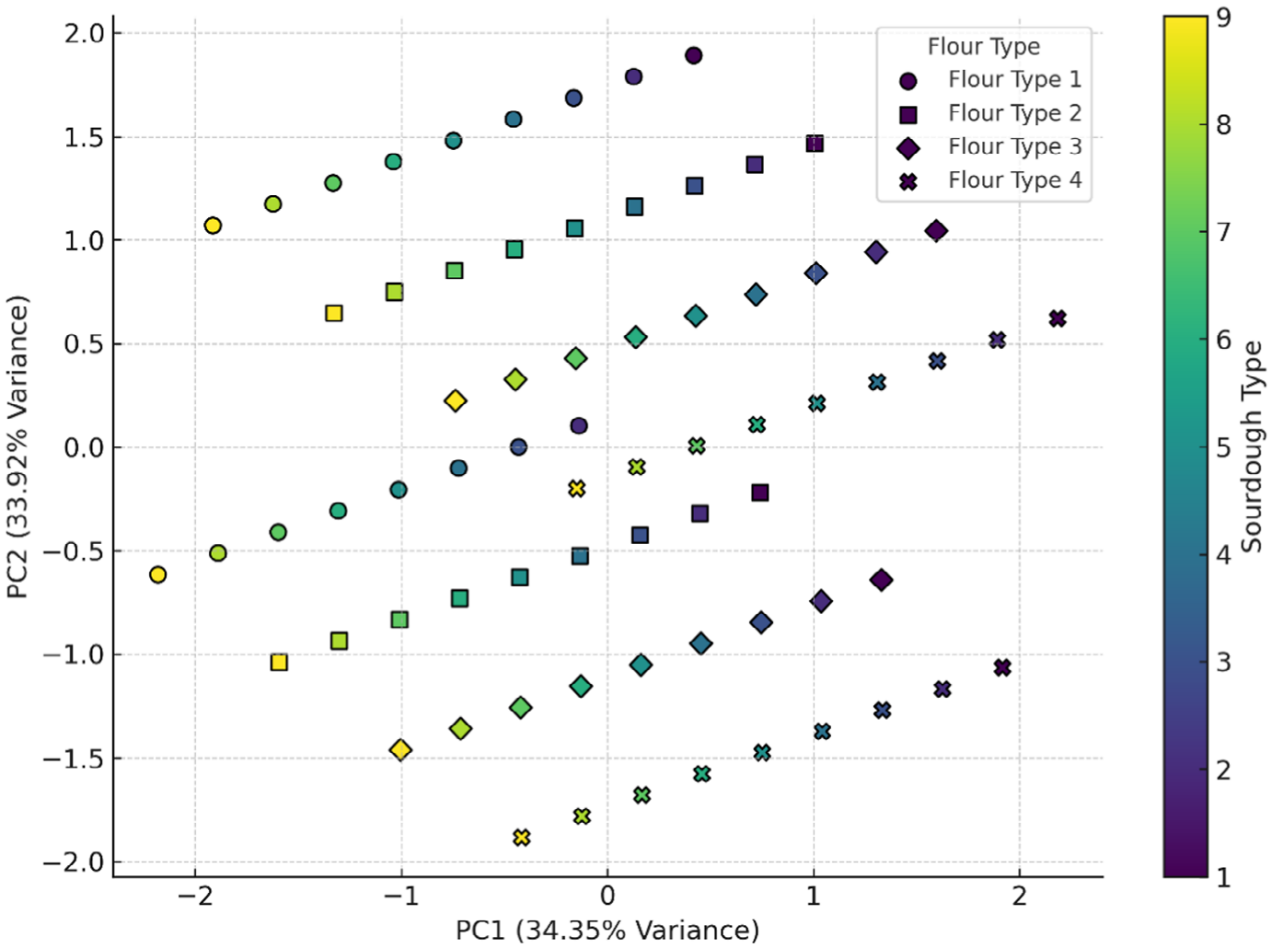
PCA analysis of the effect of different flour and sourdough combinations on Total acceptance of bread. Flour type: 1 (wheat), 2 (wheat + 5% oat), 3 (wheat + 10% oat), 4 (wheat + 15% oat); Sourdough type: 1 (wheat + *L. plantarum* ), 2 (oat + *L. plantarum* ), 3 (wheat + oat + *L. plantarum* ), 4 (wheat + *Sc. cerevisiae*), 5 (oat + *Sc. cerevisiae*), 6 (oat + wheat + *Sc. cerevisiae*), 7 ( *L. plantarum* + *Sc. cerevisiae* + wheat), 8 (oat + *L. plantarum* + *Sc. cerevisiae*), 9 (wheat + oat + *L. plantarum* + *Sc. cerevisiae*).

The sensory properties of studied bread samples, as shown in Figures 10, 11, 12, 13, 14, revealed that the most favorable combination is wheat flour with sourdough (codes 1 and 4), creating the most distinctive taste, while wheat flour + 15% oat flour with sourdough (codes 8 and 9) produced the weakest impact on flavor. Based on the odor analysis, the combination of wheat flour with Sourdough (codes 1 and 2) generally produced a better aroma in the bread. These combinations, located in the upper section of the plot, indicate a positive impact on bread odor. Wheat flour and wheat flour + 5% oat flour, along with sourdough (codes 1, 2, and 3), are clustered near the center of the plot, indicating a positive effect on bread appearance by promoting uniformity and visual appeal. PCA analysis of bread texture showed wheat flour combined with sourdough (codes 6 and 9) produced the best texture, while wheat flour + 15% oat flour alone had minimal impact, though it may benefit from pairing with Sourdough code 9. The combination of wheat flour with sourdough (code 4) achieved the highest overall acceptance score, indicating a positive impact on bread quality. Additionally, wheat flour + 10% oat flour with sourdough (code 9) and wheat flour with sourdough (code 6), both with high acceptance scores, were identified as other successful combinations. These results suggest that sourdough code 9, when combined with different flours, contributes to a high overall acceptance. The results indicate that wheat flour combined with specific sourdough codes, particularly 4 and 9, generally provides the most favorable sensory qualities across various attributes. These findings are due to bread's complex flavor profile, improved texture and crumb structure, appealing aroma, moisture retention properties, and overall enjoyable eating experience. The synergistic interactions between ingredients and fermentation processes result in a bread product that is highly satisfying and desirable to consumers (Krochmal‐Marczak, Tobiasz‐Salach, and Kaszuba 2020).

## 4 Conclusion

This study investigated the impact of different sourdough formulations and flour types on bread quality. The greatest increase in dough volume was observed in samples containing sourdough made from wheat, oat, *Lb. plantarum*, and *Sc. cerevisiae*. Oat and *Lb. plantarum* sourdough displayed the highest fiber content, while wheat and *Sc. cerevisiae* sourdough had the highest protein content. Yeast‐free breads exhibited lower porosity. Sourdoughs without *Sc. cerevisiae* exhibited elevated levels of hardness, chewiness, and cohesiveness. Yeast‐free breads showed the lowest springiness and exhibited a darker color. Sensory evaluation results revealed that breads produced with sourdough of wheat, oat, *Lb. plantarum*, and *Sc. cerevisiae* received the highest overall scores for taste, aroma, texture, appearance, and total acceptance from *assessors*. In contrast, samples lacking yeast received the lowest scores.

## Author Contributions

**Masoome Sayadi:** conceptualization (equal), formal analysis (equal), writing – original draft (equal). **Akram Arianfar:** project administration (equal), supervision (lead), writing – review and editing (equal). **Ali Mohamadi Sani:** conceptualization (equal), supervision (equal), validation (equal), writing – review and editing (equal). **Zahra Sheikholeslami:** writing – review and editing (supporting).

## Conflicts of Interest

The authors declare no conflicts of interest.

## Data Availability

The authors confirm that the data supporting the findings of this study are available within the article.
